# The efficacy and safety of Jian-Wei-Qu-Tong Pills for the treatment of chronic non-atrophic gastritis (spleen and stomach qi deficiency with damp-heat stasis syndrome): study protocol for a phase II, randomized controlled trial

**DOI:** 10.1186/1745-6215-15-272

**Published:** 2014-07-07

**Authors:** Xiao-xin Zhang, Wei-wei Chen, Bin She, Rui-jie Luo, Na Shi, Ping Xue, Xiao-nan Yang, Qing Xia

**Affiliations:** 1 Department of Integrated Traditional and Western Medicine, West China Hospital of Sichuan University, 37 Guoxue Lane, Chengdu 610041, Sichuan Province, People’s Republic of China

**Keywords:** chronic gastritis, chronic non-atrophic gastritis, spleen and stomach qi deficiency with damp-heat stasis syndrome, Jian-Wei-Qu-Tong Pills, traditional Chinese medicine, randomized controlled trial

## Abstract

**Background:**

Chronic gastritis (CG), a poorly understood entity, is a very common disease of the digestive tract and is difficult to cure. Chronic non-atrophic gastritis (CNG) is the most common type of CG. Even if treated with current standard chemotherapy, some patients will not be freed from this confusing disease. Many studies have shown traditional Chinese medicine (TCM) is more effective compared to chemotherapy in the treatment of chronic gastritis and no serious side effects have been identified. However, the studies that have been carried out were not scientifically rigorous trials. Our aim is to design a high-quality trial for a new TCM drug, the Jian-Wei-Qu-Tong Pills (JWQTP), to investigate the efficacy and safety of this new drug in treating chronic non-atrophic gastritis patients with spleen and stomach qi deficiency with damp-heat stasis syndrome (SSQDDSS).

**Methods/Design:**

This is a phase II, multicenter, parallel-group, double-blind, randomized and placebo-controlled trial. A total of 240 participants will be assigned to a high-dose group, a low-dose group or a placebo control group with a 1:1:1 ratio at five sites. Then, one dose (six 1-g pills), with a variable ratio between real drug and dummy drug according to the intervention protocol, will be taken three times a day before each meal for 8 weeks. The primary outcome is the eradication rate of epigastric pain. The secondary outcome includes the changes of endoscopic examination, histopathological examination, traditional Chinese medicine symptom scores and patient-reported outcome instrument scores for chronic gastrointestinal diseases and the eradication rate of *Helicobacter pylori* (HP).

**Discussion:**

Many CNG patients suffer from frequent, recurrent bouts of dyspeptic symptoms. This is the first clinical trial to evaluate the safety and efficacy of JWQTP in treating CNG with SSQDDSS in a multicenter, parallel-group, double-blind, randomized and placebo-controlled manner. This trial may not only provide evidence for a phase III clinical trial, but also a vision of an alternative option for CNG treatment.

**Trial registration:**

The registration number, ChiCTR-TRC-14004088, was assigned by the Chinese Clinical Trial Registry on 7 January 2014.

## Background

Chronic gastritis (CG), which is induced by various causes, is a very common disease of the digestive tract and is an inflammatory condition of the gastric mucosa [1]. Among the possible etiological factors, *Helicobacter pylori* (HP) infection is the most common cause of chronic gastritis worldwide, with the remaining cases arising due to smoking, drinking, duodenal juice reflux, food allergies, heredity, drug-induced injuries, immunity, Crohn’s disease, other infectious pathogens or radiation [2-4]. Chronic gastritis can be categorized into non-atrophic gastritis, atrophic gastritis and special types of gastritis, according to the Consensus on Chronic Gastritis in China (Shanghai 2012) [5]. Chronic non-atrophic gastritis (CNG) is characterized by the infiltration of chronic inflammatory cells and the absence of atrophy in the mucosal layer. Although the prevalence rate in the general population remains unclear, a national multicenter cross-sectional study led by the Digestive Endoscopy Branch of Chinese Medical Association showed that 49.3% of the investigated patients (4,389/8,892) with upper gastrointestinal symptoms who underwent diagnostic upper endoscopy for the evaluation of gastrointestinal symptoms from 33 centers had CNG, which was the most common [6]. Symptomatic patients with CNG may manifest nonspecific dyspeptic symptoms, such as epigastric discomfort, distention, belching, acid regurgitation, nausea, vomiting, loss of appetite and anergy, while some patients have no symptoms. The pathogenesis of chronic gastritis is complex and difficult to cure. The focused Western medical treatment of chronic gastritis involves the eradication of HP, antacids, prokinetics and mucosal-protective agents to ameliorate the symptoms [5,6]. However, even if treated with the above standard therapies, some patients will not be freed from this disease. Traditional Chinese medicine (TCM), which has recently become a research focus in particular contexts, can broaden the therapeutic approaches to CNG [7-11].

Traditional Chinese medicine, which has been established for over 5,000 years, has been widely used to treat diseases, including CNG. Based on its clinical manifestations, CNG can be categorized as Weiwantong (stomach ache), Piman (abdominal distention) or Caoza (gastritis discomfort) in the field of Chinese medicine (CM); TCM divides CNG into five common single syndrome patterns: (1) spleen (Pi) and stomach (Wei) deficiency syndrome (including spleen and stomach qi deficiency syndrome and spleen and stomach deficiency-cold syndrome); (2) incoordination between liver (Gan) and stomach syndrome (including liver qi invading stomach syndrome and stagnant heat in liver and stomach syndrome); (3) stomach yin deficiency syndrome; (4) spleen and stomach dampness-heat syndrome; and (5) blood stasis in stomach collaterals syndrome [3]. The concurrence of two or more individual patterns can be considered a complicated pattern and is common in clinical practice [12]. The TCM treatment relies on correct syndrome differentiation (SD), by which accumulating symptoms and signs are identified through inspection, auscultation and olfaction, inquiring, and palpation to uncover the ongoing abnormal condition. Therefore, TCM agents operate through other pathways that may be helpful for patients who are nonresponsive to standard therapies.

A new TCM drug, the Jian-Wei-Qu-Tong Pills (JWQTP), is currently being manufactured by Anbang Pharmaceutical Limited by Share Ltd, Hunan, China. The prescription for this pill is based on both TCM theory and the clinical experience and herbal studies of prominent TCM doctors on the treatment of CNG. Components of the pill include: Radix Pseudostellariae, Rhizoma Coptidis, Rhizoma Atractylodis Macrocephalae, Semen Coicis, Rhizoma Pinelliae Preparata, Radix Notoginseng, Pollen Typhae, Herba Taraxaci Mongolici, Rhizoma Corydalis Yanhusuo, Cortex Magnoliae Officinalis and Radix Glycyrrhizae preparata. All of these herbs have been approved by the China Food and Drug Administration (CFDA). This pill can strengthen the spleen, supplement qi, clear heat, remove dampness and promote blood circulation to remove blood stasis and relieve pain. Thus, it can be used for spleen and stomach qi deficiency with damp-heat stasis syndrome (SSQDDSS) in CNG patients. Preclinical pharmacologic experiments, which have not been published, show that JWQTP can neutralize gastric acid; reduce gastric acidity, pepsin activity, free mucus, gastrin, TNF-α and IL-2; elevate the level of IL-4; create balance between the Th1 and Th2 cells; and then reduce inflammation in rats’ gastric mucosa to protect against CG. Furthermore, this treatment was able to eradicate HP in the gastric mucosa of mice to some degree, provide an analgesic effect and play a regulatory role on gastric emptying. Additionally, there was no evidence to show an adverse or toxic effect in toxicological studies.

The specific objectives of this trial included evaluating the efficacy and safety of JWQTP in treating CNG patients with SSQDDSS, including any dose-effect relationship, and providing evidence for a Phase III clinical program.

## Methods/Design

**Figure 1:**
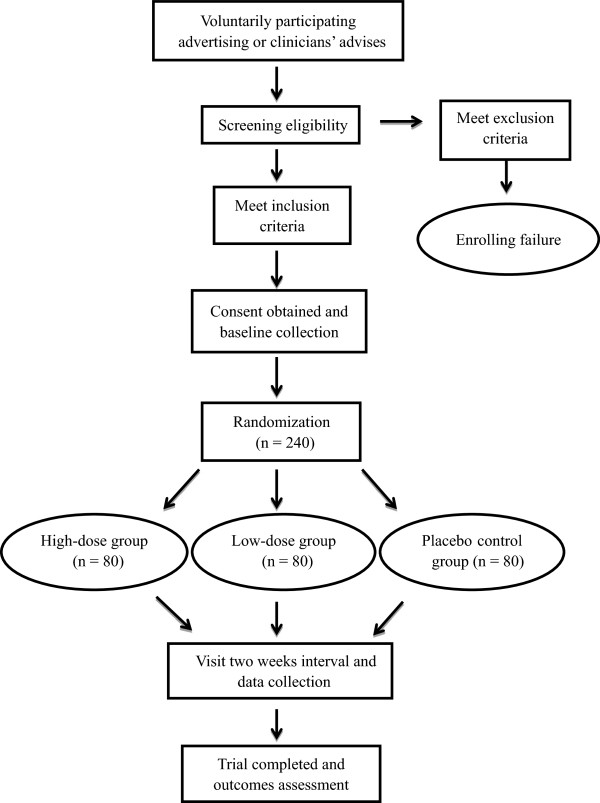
Study flow chart.

This current study is a phase II, multicenter, parallel-group, double-blind, randomized and placebo-controlled trial. The study will be conducted in compliance with the Declaration of Helsinki, Good Clinical Practice (GCP) Guidelines [13] and the requirements of clinical trials by the Drug Administration Law of the People's Republic of China, and will strictly observe all laws governing new TCM drugs. The study was approved by the CFDA (Approval No. 2012 L02382), and the protocol and informed consent were reviewed and approved by the West China Hospital of Sichuan University Clinical Trials and Biomedical Ethics Committee (No. TCM-2013-06). In addition, it was registered in the Chinese Clinical Trial Registry (ChiCTR-TRC-14004088). We will recruit subjects by advertising on the hospital’s notice board or through recommendations in the outpatient clinic. All patients must provide written informed consent. The trial is financially supported by Anbang Pharmaceutical Limited by Share Ltd, Hunan, China, which did not contribute to the study design, data collection, data management, analysis, interpretation of data or decision to submit the report for publication, except for the provision of all test drugs. Each trial center has a project manager who is responsible for the quality of research. Standard training will be required for all investigators before the trial. An independent trial inspector will pay regular visits to each center, check the case report forms (CRFs) and supervise the research to make sure it complies with the protocol throughout the trial. Figure 1 shows the flow chart of this trial.

### Patient population and setting

A total of 240 patients will be enrolled at the following five sites: (1) West China Hospital of Sichuan University, (2) the first Affiliated Hospital of Guiyang College of Traditional Chinese medicine, (3) the first Affiliated Hospital of Hunan University of Chinese Medicine, (4) the first Affiliated Hospital of Guangxi University of Chinese Medicine and (5) the Ruikang Hospital affiliated to Guangxi University of Chinese Medicine. In other words, 48 patients will be recruited at each hospital.

### Randomization

For randomization, the participants will be assigned into a high-dose group, a low-dose group or a placebo control group, with a 1:1:1 ratio using a stratified randomization method, with stratification by center. The randomization sequence will be created at the West China Hospital of Sichuan using SAS 9.2 software to determine the appropriate segment. Two copies of the randomization list will be kept in safe places - one by the West China Hospital of Sichuan University drug clinical trial agency and one by the sponsor of this study - during the study period. Each patient will receive a unique randomized test number corresponding to the specified drug, according to the group allocation. An emergency envelope has been prepared for each test number and is to be opened for treatment allocation only due to medical emergency.

### Diagnosis in Western medicine

Referring to consensus on chronic gastritis in China (Shanghai, 2012) [5], consensus on the diagnosis and treatment of chronic gastritis by integrative medicine (Tianjin 2011) [14], Practice of Internal Medicine (2009, Version 13) [15] and endoscopic classification and trial standards on treatment of chronic gastritis (Dalian, 2003) [16], which is the diagnostic criteria for CNG in Western medicine, are listed as follows:

#### Clinical manifestations

Most symptomatic patients with CNG have nonspecific dyspepsia, such as epigastric pain, distention, belching, acid regurgitation, nausea, vomiting and loss of appetite.

#### Physical signs

There is no finding in most patients’ physical examinations, although some patients may feel slight epigastric pain or discomfort when pressed on the upper abdomen. Only a tiny minority of these patients are gaunt or anemic.

#### Endoscopic and histopathological diagnosis

Because there is no specific symptom, the diagnosis of CNG relies primarily on endoscopic and histopathological examinations, particularly the latter. The fundamental appearance of non-atrophic gastritis shows red plaques (punctuates, patches and striae), coarse and uneven mucosa, hemorrhagic spots/plaques, edematous mucosa, and exudates at endoscopy. No atrophic changes, intestinal metaplasia, dysplasia or pseudopyloric gland metaplasia are found in the histopathological examination of the gastric mucosa. Table 1 shows that the red plaques, erosion, hemorrhage and bile reflux are further graded into four grades at endoscopy, as chronic inflammation and active chronic inflammation in the histopathological examination observed are in Table 2.

**Table 1:** Endoscopic characteristics, grading and scoring

**Endoscopic appearance**	**Endoscopic characteristics**	**Grading system**	**Score**
**Red plaques**	Apparent rubefaction in the normal gastric mucosa	None	0
		Grade I: sporadic or interrupted linear type	1
		Grade II: intensive plaques or linear series type	2
		Grade III: extensive fusion	3
**Erosion**	Flattened or varioliform erosions: superficial mucosa is ruptured, along with flat or ridged erosion in the surrounding area	None	0
		Grade I: sporadic	1
		Grade II: local, multiple but no more than five	2
		Grade III: extensive, multiple and no less than six	3
**Hemorrhage**	Hemorrhage in the mucosa: flattened hemorrhagic spots/plaques with bright red or dark red, together or not with errhysis	None	0
		Grade I: local	1
		Grade II: multiple sites	2
		Grade III: widespread	3
**Bile reflux**	Olive green gastric juice; bile flow back into stomach because of opened pyloric orifice	None	0
		Grade I: yellowish or flavo-green mucinous lake	1
		Grade II: lies between grade I and grade III	2
		Grade III: massive yellow bile flow back into stomach, yellow mucus adhere to gastric mucosa	3

**Table 2:** **Histopathological changes, grading and scoring**^**a**^

**Histopathological changes**	**Grading system**	**Score**
**Chronic inflammation**	Normal or number of mononuclear cells less than 5/high power field	0
	+ (mild): Some chronic inflammatory cells are localized in the superficial layer of the mucosa, but not more than 1/3 of the depth of the mucosa;	1
	++ (moderate): A more dense accumulation of chronic inflammatory cells, but not exceeding 2/3 of the depth of the mucosa	2
	+++ (severe): A dense accumulation of chronic inflammatory cells occupying the whole depth of the mucosa	3
**Active chronic inflammation**	Normal	0
	+ (mile): Few neutrophils in the lamina propria of the mucosa	1
	++ (moderate): More neutrophils in the mucosal layer and between superficial epithelial cells, pit epithelial cells and glandular epithelial cells.	2
	+++ (severe): A more dense infiltration of neutrophils, or abscess on pits can be seen in addition to what is seen in moderate activity.	3

^**a**^Two to five biopsy specimens reaching musculature in the mucosa will be taken from gastric antral, gastric angle or suspected focal lesions of sufficient size. For the specimens taken at the first and sixth visits, the regions should be chosen consistently as possible as it can.

### Chinese medicine diagnostic criteria of the spleen and stomach qi deficiency with damp-heat stasis syndrome

In acknowledgement of the consensus on diagnosis and treatment of chronic superficial gastritis with TCM (Shenzhen, 2009, Digestive Disease Branch of China Association for Traditional Chinese Medicine) [17], The People’s Republic of China TCM industry standards • the standards of diagnosis and efficacy of TCM syndromes [18], and the *Guidelines For Clinical Research On Chinese New Herbal Medicines (Trial Implementation)*[19], the CM diagnostic criteria of SSQDDSS in CNG patients are listed and include the following:

1. Primary symptom: epigastric pain.

2. Secondary symptoms: weakness and lassitude, dry mouth/bitter taste in mouth, loss of appetite, distention and fullness, loose stool or belching.

3. Tongue presentations: pale tongue, dark red tongue with ecchymosis or petechiae or enlarged and teeth-printed tongue.

4. Tongue coating: yellow or yellowish greasy coating.

5. Pulse presentations: thread and weak pulse or stringy pulse.

A diagnosis of SSQDDSS should include the primary symptom, the first two secondary symptoms and more than one of the remaining four secondary symptoms, simultaneously combined with tongue presentations and pulse presentations.

### Inclusion criteria

Inclusion criteria are:

1. Diagnosis of CNG according to Western medicine (moderate or severe level of chronic inflammation and/or active inflammation).

2. Diagnosis of spleen and stomach qi deficiency with damp-heat stasis syndrome, according to TCM syndrome differentiation.

3. Aged between 18 and 65 years.

4. Voluntary provision of written informed consent prior to enrollment.

### Exclusion criteria

Exclusion criteria are:

1. History of gastric surgery.

2. Combined gastric ulcer, duodenal ulcer, special types of gastritis or gastrointestinal hemorrhage.

3. Histopathological examination showing atrophic changes, intestinal metaplasia, dysplasia or suspected malignant changes in the gastric mucosa.

4. Serious comorbidities of heart, lung, liver, kidney or blood system (such as cardiac function above grade II, alanine aminotransferase (ALT) and/or aspartate aminotransferase (AST) greater than 1.5 times the upper limit of normal, creatinine (Cr) greater than the upper limit of normal and so on) or having a life-threatening illness (for example, tumor or AIDS).

5. Positive fecal occult blood test.

6. Having received TCM or Western medicines to treat CNG in the last 2 weeks.

7. Psychiatric disorders or a history of alcohol or drug abuse.

8. Female patients preparing for a baby, pregnant or lactating.

9. Allergic to the trial pills.

10. Currently participating or enrolled in another drug trial over the last three months.

11. Unclear TCM diagnosis of syndrome differentiation or too complicated SDs in TCM.

12. Having been judged inappropriate to participate in the trial by investigators.

### Rejection criteria

Rejection criteria include:

1. Misdiagnosis.

2. The trial drug is taken at doses <80% or >120% of the requirement, or is not taken as directed.

3. Patients do not cooperate or cannot complete follow-up.

### Withdrawal criteria

Withdrawal criteria include:

1. Intolerance to worsening conditions.

2. Quitting voluntarily.

3. Having manifestations of hemorrhage of the digestive tract, such as melena or hematemesis during the study.

4. Experiencing anaphylaxis following the trial drug’s administration.

5. Adverse medical events occurring during the study, such as severe liver function damage, malignant tumors in other system, and accidental pregnancy or other reasons, as judged by the investigators, that might make the patient unlikely to complete the study.

### Suspending early or terminating the entire study

The study may be suspended early or terminated in the event of the following:

1. Serious adverse event (SAE) caused by the trial drugs.

2. The effect of the test drug is poor or even completely ineffective.

3. Discovery of a blunder in the protocol or of a significant deviation between the protocol and actual practice

4. The decision to terminate the study being made by the pharmaceutical supervisory and administrative department or by the sponsors, for any reason.

### Concomitant treatments and forbidden drugs

Subjects will be permitted to continue concomitant treatment for comorbidities, such as hypertension, chronic bronchitis, stroke or skin disease, which will not affect the trial’s final results. However, they are forbidden to take any CM or Western medicine related to CNG, such as prokinetics, cholinergic agents, H2-receptor antagonists, proton pump inhibitors, mucosal-protective agents or antibiotics for the eradication of HP. Aspirin, nonsteroidal anti-inflammatory drugs, sedative hypnotics, antidepressants and opioids should be used sparingly. Each concomitant treatment should be documented strictly in the CRF, and once a subject is found to be taking forbidden drugs, he/she will be withdrawn from this study.

### Test drugs and blinding

**Figure 2:**
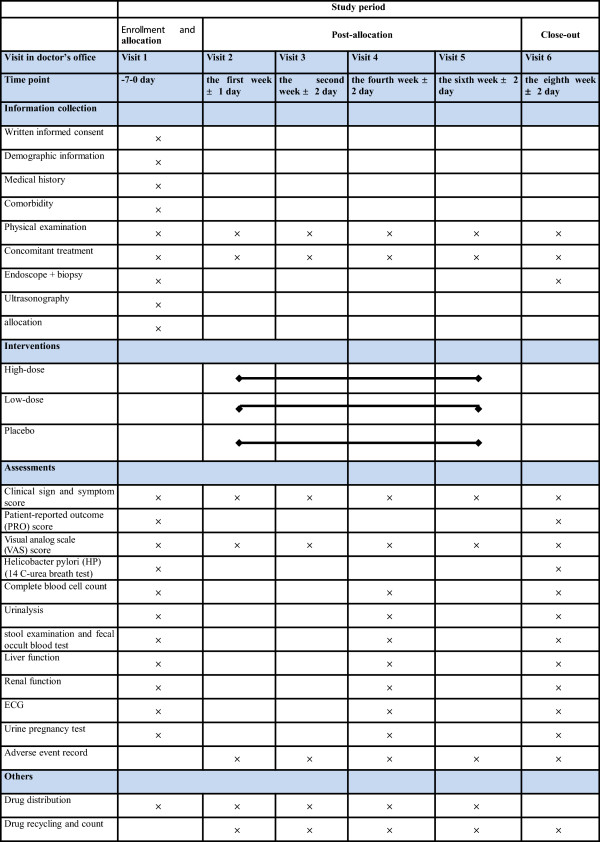
Study schedule for patients.

The JWQTP and the dummy JWQTP will be manufactured by Anbang pharmaceutical Limited by Share Ltd, Hunan, China, with the approval of the CFDA for clinical studies only (Approval No. 2012 L02382). Of note, the dummy JWQTP is not exactly the same as the test pill in color and smell. To make a blind test possible, all of the drugs are concealed in unified, sealed and opaque packages with the same labels that contain the drug name, the approval number of the pill, functions, usage, dosage, storage conditions, expiration dates for use and the manufacturer’s name. The drugs are administered by an independent clinical assistant in each center, who takes responsibility for the drug’s distribution, storage and return. Any members who have access to the drug will not participate in case observations or efficacy evaluations. Each package contains one dose (six 1-g pills) with a variable ratio between the real drug and the dummy drug, according to the intervention protocol. Patients will be randomized to a high-dose group, low-dose group or placebo control group, in which they will take 6 g real drugs, 4 g real drugs + 2 g dummy drugs or 6 g dummy drugs, respectively. All drugs will be taken orally with water before each meal three times a day for 8 weeks. A follow-up visit will be scheduled every 2 weeks after treatment. Patients who have not made a full recovery from CNG after the clinical study will receive conventional treatment. Details of the procedures and content are shown in Figure 2.

### Outcome measurement

#### Primary outcome

The eradication of epigastric pain is considered the primary outcome to evaluate therapeutic effects. The visual analogue scale (VAS) score (0 to 10 numeric pain intensity scale) for epigastric pain was measured at every visit, ranging from 0 to 10, where a score of 0 suggests no epigastric pain and a score of 10 indicates the most severe epigastric pain. The eradication of epigastric pain is defined as a score of VAS that stops rising after dropping to 0 during the study. The eradication rate of epigastric pain will be compared among the three groups, together with the change in VAS scores before and after treatment in each group.

#### Secondary outcomes

##### Endoscopic and histopathological examination

The change in red plaques, hemorrhagic spots/plaques, erosion and bile reflux at endoscopy and the grade of active chronic inflammation and chronic inflammation in the gastric mucosa, depending on the histopathological examination of biopsy specimens, will be compared between visits one and six, according to the consensus on CG in China.

##### Eradication rate of Helicobacter pylori

The eradication rate of HP after treatment will be used to evaluate the effects of JWQTP.

##### The treatment efficacy in the Chinese medicine field

To evaluate the therapeutic effect in the CM field, the TCM symptom score system (Table 3) will be used, following the *Guidelines for Clinical Research on Chinese New Herbal Medicines* (*Trial Implementation*). In the symptom score system, the score of the primary symptom will be based on the severity, duration and frequency of the symptoms. Secondary symptoms will be scored by grading. The final point will be determined by the sum of all symptom scores. The CM therapeutic effect Index (CMTEI), as calculated with the following formula, will be used to evaluate treatment efficacy in the CM field.

**Table 3:** **The traditional Chinese medicine (TCM) symptom score system for chronic non-atrophic gastritis (CNG**)

**Symptom**			**Score**	**Grading system**
**Primary symptom**	Epigastric pain	Degree		0 score: None
				2 score: Mild pain, does not affect work and life, VAS score 1 to 3
				4 score: Moderate pain, affects work and life partly, VAS score 4 to 6
				6 score: Severe pain, affects work and life fully, need rest, VAS score 7 to 10
		Duration		0 score: None
				2 score: Less than 1 hour a day
				4 score: 1 to 2 hours a day
				6 score: More than 2 hours a day
**Total primary symptom scores**				Degree score + Duration score
**Frequency of epigastric pain**				Score = the days of pain attack in a week (for example, a score of 7 indicates pain everyday)
**Secondary symptoms**	Weakness and lassitude			0 score: No
				2 score: Yes
	Dry mouth/bitter taste in mouth			0 score: No
				2 score: Yes
	Loss of appetite			0 score: None
				1 score: food intake reduced by 1/3
				2 score: food intake reduced by 1/3 to 2/3
				3 score: food intake reduced by 2/3
	Distention and fullness			0 score: None
				1 score: Mild,
				2 score: Moderate, does not affect work and life
				3 score: Severe, need drug control
	Loose stool			0 score: No
				2 score: Yes
	Belching			0 score: No
				2 score: Yes
**Total symptom score**				Total symptom score = total primary symptom scores + total secondary symptom scores. Frequency of epigastric pain score is calculated independently.

$$\begin{array}{l} \mathrm{CMTEI}=\\ \left(\frac{\mathrm{symptom}\;\mathrm{score}\;\mathrm{before}\;\mathrm{treatment}‒\mathrm{symptom}\;\mathrm{score}\;\mathrm{after}\;\mathrm{treatment}}{{}_{*100\%}\;\mathrm{symptom}\;\mathrm{score}\;\mathrm{before}\;\mathrm{treatment}}\right) \end{array}$$

The classification of treatment efficacy is shown as follows: CMTEI ≥95% indicates clinical recovery; 95% > CMTEI ≥70% indicates remarkable recovery; 70% > CMTEI ≥30% indicates modest recovery; and CMTEI < 30% indicates inefficiency.

#### The change in the patient-reported outcome instrument for chronic gastrointestinal diseases

Although the symptoms of chronic gastritis are not specific, the relief of clinical symptoms remains a major therapeutic aim. The patient-reported outcome (PRO) instrument for chronic gastrointestinal diseases (http://www.proqolid.org/) [20], a 6-dimension, 35-item instrument, will be performed on visits one and six, and the results will be used to compare the two visits to assess any changes in quality of life. The 35 items of the instrument are the following: fatigue, disturbed sleep, no hunger sensation, decreased appetite, afraid of society, dry mouth or bitter taste in mouth, bad breath, pharyngeal foreign body sensation, acid regurgitation, hiccup or belching, nausea or vomiting, burning pain behind the sternum, retrosternal pain, stomachache, gastric distension, gastric discomfort, stomach burning, abdominal pain, abdominal distension, degree of retrosternal pain, degree of heartburn, degree of stomachache, degree of gastric distension, degree of abdominal pain, degree of abdominal distension, diarrhea, constipation, incomplete sensation after defecation, defecation urgency, weight loss, emotional fluctuation, anxiety or nervous, worry about disease, impact on social activities and impact on family status and function. All of the items fall into five levels, which are scored as 0, 1, 2, 3 or 4, except for decreased appetite, which is grouped into four levels and scored as 0, 1, 2 or 3. The final score will be determined by the sum of all symptom scores.

#### Safety outcomes

We will perform the following tests on all subjects to screen for the JWQTP administration during the study: physical examination (temperature, respiration, heart rate, blood pressure, height and weight), complete blood cell count, urinalysis, stool examination, fecal occult blood test, liver function (ALT, AST, alkaline phosphatase (ALP), serum total bilirubin (STB), and γ-glutamyl transpeptidase (γ-GT)), renal function (Cr, micro albuminuria, serum cystatin C, and urine N-acetyl-β-glucosaminidase) and electrocardiogram (ECG) at the first, fourth and sixth visits. In addition, a urine pregnancy test will be carried out for female patients of childbearing age (Figure 2).

#### Data management

The completed CRFs will be submitted to the statistical department after the inspections of the clinical investigators and trial inspectors for data entry and management. All data will be entered twice electronically using the EpiData3.1 database by two independent data managers to ensure data accuracy. The investigators will resolve any problems encountered by the data managers’ concerning the data as soon as possible. Then, the data managers will make corrections to the data, according to the investigators’ instructions. A blind review will be performed among the data managers, predominant investigator and sponsor to compile a report about the quality of data management. The database will be locked by the statistician, predominant investigator and sponsor after the blind review and confirmation of database accuracy. Then, the final trial dataset will be disclosed by an independent assistant according to the randomization list, where the data will be grouped into groups A, B or C, and an independent statistician will make statistical analysis using SAS 9.2 in a semi-blinded manner with no suggestion of the exact treatment allocation. Finally, the predominant investigator will write a conclusive report of the statistical analysis in an unblinded manner. Because there will be unified training prior to the study, regular supervisor’s visits, data review by the clinical investigators, revisions by the data managers and a blind review for the data after the study, no further data management committee will be set up to ensure the data’s accuracy and validity. Only the clinical investigators and trial inspector will have access to the medical records of the subjects, and they will all sign a confidentiality agreement. Anonymous data will be used for the inspection of the pharmaceutical supervisory and administrative department. Subjects’ medical records will be stored in the data archive of the drug clinical trial agency after analysis.

#### Adverse event reporting

The safety of subjects is of paramount importance during the study period, and every adverse event should be documented on the adverse event form (AEF) in great detail. Adverse events (AEs) include any new diseases and exacerbations of CNG or comorbidities related or unrelated to the treatment. Any fatal, life-threatening, disabling or event severe enough to warrant hospital admission or prolonged hospital stays occurring after any dose of treatment pill and at any time during this study should be considered an SAE. All of these data should be recorded on an AEF with the corresponding treatment and reported to the China Food and Drug Administration (CFDA), provincial-level Food and Drug Administration, sponsor and Independent Ethics Committee within 24 hours. Then, the sponsor will inform the other four sites as soon as possible. The severity of an AE is classified into three degrees: mild, moderate and severe. For the mild degree, a small amount of indisposition arises, which the subject can bear without any intervention and without influencing the trial. For the moderate degree, a moderate amount of indisposition develops, and some intervention should be taken to get the trial going. For the severe degree, an enormous amount of indisposition dictates that the subject should quit the trial for his or her own safety. In the case of an SAE, the investigator can break the blind to discover the subject’s treatment allocation by reading the emergency letter corresponding to the group allocation. Any AE related to the test drug will be treated for free. On a separate note, any subject with an intervention for AE must undertake a follow-up visit within half a month for his or her health.

#### Sample size calculation

Based on the preliminary results of clinical research, no less than an estimated 55% total efficiency will be obtained with JWQTP treatment and approximately 30% will be obtained with the intervention of a placebo. Using a superiority test, no fewer than 66 subjects should be enrolled based on α = 0.05, β = 0.15 and power = 0.85 in each treatment group. Allowing for a 20% drop-out, we therefore propose the recruitment of a total sample size of 240 patients, with average distributions among the three groups.

#### Statistical analysis

A full analysis set (FAS) is the primary analysis population for efficacy with an intention-to-treat principle. We estimate the missing data using the last observation carried forward (LOCF) method so that the population for the final analysis corresponds with that at the beginning of this study. The per-protocol set (PPS), in which all subjects enable themselves to finish the study according to the protocol, represents a subcardinal analysis population for efficacy. Descriptive statistics will be presented as the means, standard deviations, medians, minimum values and maximum values for continuous variables and as frequencies and percentages for categorical variables.

An analysis of variance (ANOVA) or chi-square test will be used to compare baselines among patients, including demographic data, vital signs, history of diseases, basic treatment and so on. For the comparison of variations from the baseline to endpoint, Student’s t-test will be performed on variables with normal distributions, and the Wilcoxon rank sum test will be performed on variables with non-normal distributions. The evaluation of therapeutic effects will primarily be performed between baseline and visit six, in compliance with PPS, or the time to withdraw from the study, in compliance with FAS. In addition, the changes from baseline to visits two, three, four or five will be evaluated. For continuous variables regarding therapeutic effect, an ANOVA will be used for multiple comparisons of variation from baseline among different groups, and Dunnett’s tests will be used for comparisons within a group. A covariance analysis, adjusted for the baseline covariate, will be used to evaluate the differences of the symptom scores, VAS scores and PRO instrument scores among the groups considering the center effect. The least square mean in each group and the 95% confidence intervals of mean differences among groups will be calculated. The Cochran-Mantel-Haenszel test, stratified by center effect, and the logistic regression model, adjusted for covariates, will be used on categorical variables in addition to the chi-square test, Wilcoxon rank sum test or Fisher’s exact test. The total drop-out rate in each group will be compared with the drop-out rate due to AEs for drop-out analyses using chi-square test or Fisher’s exact test. For safety analyses, we will perform the chi-square test or Fisher’s exact test to evaluate the incidence rate of AEs among the groups and describe AEs using descriptive statistics through listing.

All statistical tests will be performed by an independent statistician using SAS 9.2, assuming a two-sided test and a 0.05 level of significance.

## Discussion

To date, CNG remains unclear and has no specific remedy. The presence or absence and severity of dyspepsia have no obvious correlations with the histopathological findings and endoscopic grading of CG [3]. The therapeutic aim of CNG is to ameliorate these symptoms and to reduce the inflammation of the gastric mucosa, mainly focusing on the eradication of HP, prokinetics, antacids (H2-receptor antagonist or a proton pump inhibitor), mucosal-protective agents, anti-depression drugs and anti-anxiety drugs [5]. However, many CNG patients suffer from recurrent bouts of dyspeptic symptoms because these symptoms are easily influenced by psychological, social or dietary factors. TCM was more effective than chemotherapy in the treatment of chronic gastritis, including all subtypes, and no serious side effects have been identified. TCM has been widely considered an alternative option for chronic gastritis in East Asia [7,21]. According to the present findings, no scientifically rigorous trials have been designed to treat CNG patients with SSQDDSS. This study will be the first to explore the efficacy and safety of JWQTP and to provide evidence and an optimal dosage for a phase III clinical trial.

## Trial status

This trial is preparing for recruitment of participants at the time of manuscript submission.

## Abbreviations

AE: adverse event; AEF: adverse event form; AIDS: acquired immunodeficiency syndrome; ALP: alkaline phosphatase; ALT: alanine aminotransferase; ANOVA: analysis of variance; AST: aspartate aminotransferase; CFDA: The China Food and Drug Administration; CG: chronic gastritis; CM: Chinese medicine; CMTEI: Chinese medicine therapeutic effect index; CNG: chronic non-atrophic gastritis; Cr: creatinine; CRF: case report form; ECG: electrocardiogram; FAS: full analysis set; HP: *Helicobacter pylori*; JWQTP: Jian-Wei-Qu-Tong Pills; LOCF: last observation carried forward; PPS: per-protocol set; PRO: patient-reported outcome; SAE: serious adverse events; SD: syndrome differentiation; STB: serum total bilirubin; SSQDDSS: spleen and stomach qi deficiency with damp-heat stasis syndrome; TCM: traditional Chinese medicine; VAS: visual analog scale; γ-GT: γ-glutamyl transpeptidase.

## Competing interests

The authors declare that they have no competing interests.

## Authors’ contributions

XX Z, WW C and QX participated in the planning, design and development of this trial. The trial will be performed under the careful supervision of QX, who will oversee the trial during the study. XX Z and WW C drafted the initial manuscript with equal contribution and should be considered co-first authors. RJ L, NS and PX contributed to the conception and design of the protocol and revised the manuscript critically for important intellectual content. BS and XN Y participated in the design and coordination of the study, as well as recruiting participants. All authors have reviewed and approved the final manuscript.
